# The TAM-TB Assay—A Promising TB Immune-Diagnostic Test With a Potential for Treatment Monitoring

**DOI:** 10.3389/fped.2019.00027

**Published:** 2019-02-11

**Authors:** Mohamed I. M. Ahmed, Christian Ziegler, Kathrin Held, Ilja Dubinski, Julia Ley-Zaporozhan, Christof Geldmacher, Ulrich von Both

**Affiliations:** ^1^German Center for Infection Research (DZIF), Partner Site Munich, Munich, Germany; ^2^Department of Tropical Medicine and Infectious Diseases, University Hospital, Ludwig-Maximilians-University, Munich, Germany; ^3^CIH^*LMU*^ Center for International Health, University of Munich, Munich, Germany; ^4^Division of Orthopaedics, Ludwig-Maximilians-University, Munich, Germany; ^5^Division of Paediatric Infectious Diseases, Dr. von Hauner Children's Hospital, University Hospital, Ludwig-Maximilians-University, Munich, Germany; ^6^Department of Radiology, Paediatric Radiology, University Hospital, Ludwig-Maximilians-University, Munich, Germany; ^7^Section of Paediatric Infectious Diseases and Allergy, Department of Medicine, Imperial College London, London, United Kingdom

**Keywords:** extra-pulmonary tuberculosis, treatment monitoring, TAM-TB assay, TB diagnostics, pediatric tuberculosis

## Abstract

Tuberculosis (TB) epidemiology is changing in Western and Central Europe due to the rise in immigration and refugees fleeing high-TB-burden areas of war and devastation. The change in local demography and the lack of sensitive and specific TB diagnostic and monitoring tools, especially for cases of childhood TB, leads to either missed cases or over-treatment of this group. Here we present a promising new diagnostic approach, the T cell activation marker (TAM)-TB assay, and its performance in a case of extra-pulmonary TB occurring in a 16 year old refugee from Afghanistan. This assay is based on the characterization of 3 activation markers (CD38, HLA-DR, and Ki67) and one maturation marker (CD27) on *M. tuberculosis*-specific CD4 T cells. It was performed at time-points T0 (10 days), T1 (1 month), T2 (6 months), and T3 (12 months) post-treatment initiation. All markers were able to detect active tuberculosis (aTB) within this patient at T0 and reverted to a healthy/LTBI phenotype at the end of treatment. Tantalizingly, there was a clear trend toward the healthy/LTBI phenotype for the markers at T1 and T2, indicating a potential role in monitoring anti-TB treatment in the future. This assay may therefore contribute to improved TB diagnostic algorithms and TB treatment monitoring, potentially allowing for individualization of TB treatment duration in the future.

## Introduction

Childhood TB, particularly in its extra-pulmonary form, is very challenging to diagnose, let alone to monitor treatment response (1, 2). Predictive markers that would allow clinicians to differentiate between ongoing active disease and cure as well as specific correlates for protection are lacking (3). Thus, there is an urgent need for improved diagnostic tests. Traditional diagnostic tests such as the Tuberculin Skin Test (TST) or the Interferon Gamma Release Assays (IGRAs) are primarily detecting *Mycobacterium* tuberculosis (MTB) infection, however their inability to differentiate between aTB, LTBI and successfully treated TB makes them an unsatisfactory tool for diagnosis of active TB. Only two diagnostic methods in use form the current “gold standard,” the liquid culture method, and the PCR technique (GeneXpert MTB/RIF^®^, Cepheid) both of which involve the detection of MTB in sputum or other clinical samples. However, the reliance on sputum samples makes the liquid culture susceptible to contamination (4). Furthermore, obtaining sputum samples from pediatric patients, especially in infants, is difficult. Another, group of patients where microbiological detection of MTB tends to be difficult consists of extra-pulmonary cases who do not necessarily produce sputum containing MTB. In addition to the described sampling issues the liquid culture method is a long process, requiring weeks for results to be available, Furthermore, a major limitation of all currently available diagnostic tools is their inability to monitor treatment response. Liquid culture methods require weeks for results to be available, and as treatment progresses the production of sputum and detection of live MTB in sputum becomes more difficult (5). The GeneXpert MTB/RIF^®^ method, though being a very sensitive method is hampered by its inability to differentiate between live and dead bacilli, thus not allowing for evaluating success of treatment (6). This has led to a presumed overtreatment of a majority of pulmonary TB patients, shown in previous studies reporting that at least 80% of pulmonary TB patients had been cured within 4 month of anti-TB treatment not showing any signs of relapse within the defined study period (7–9). An optimal monitoring tool would be rapid and dynamic to allow *in vivo* measurement of changes of biomarkers to mirror treatment progress in each individual patient, this opens the avenue toward a personalized medicine approach in TB care—particularly in children and adolescents.

## An Exemplary Case

**Figure 1:**
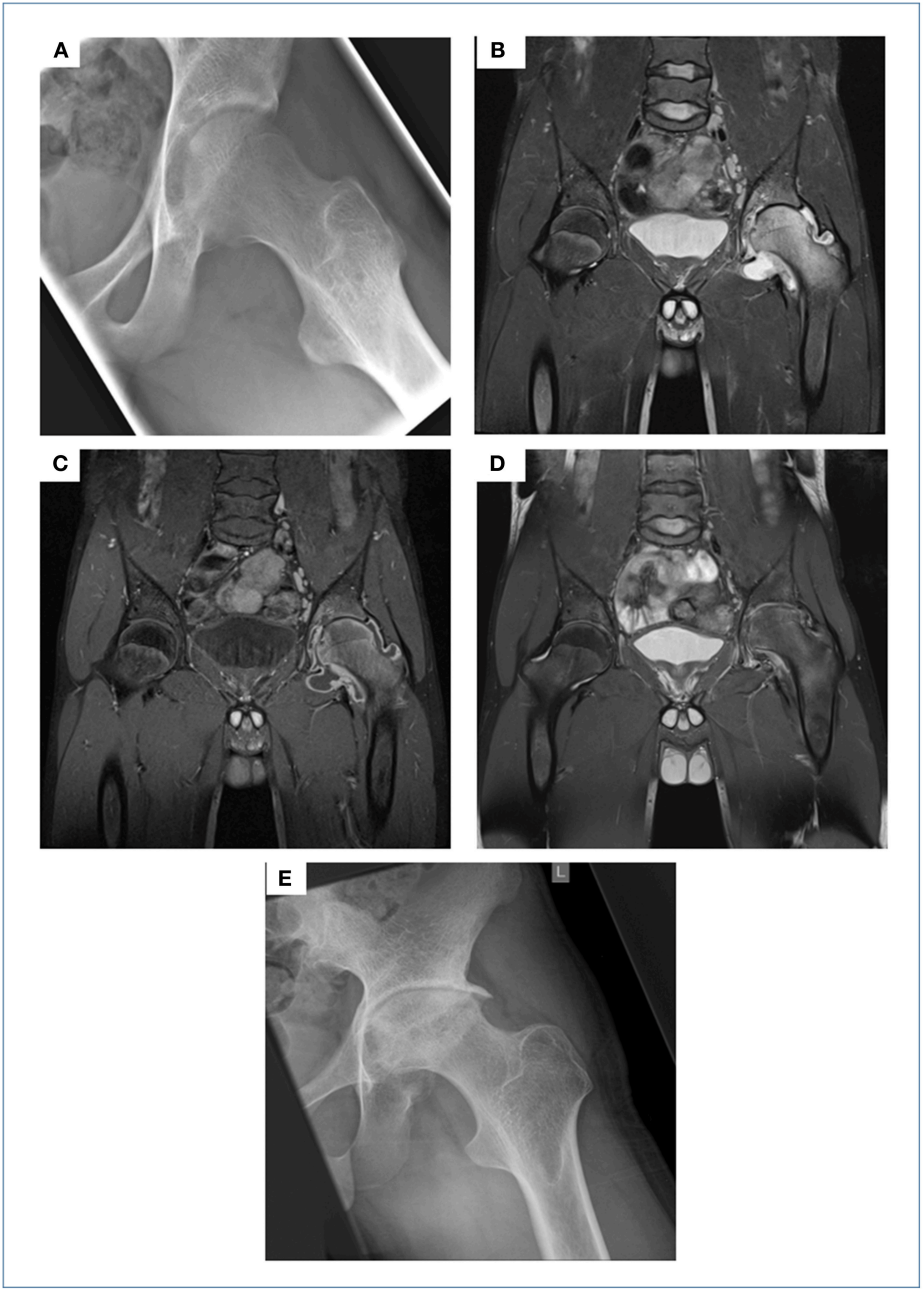
Initial X-ray of the left hip showed blurring of the cortical margins of the femoral head and acetabulum, slight demineralization, and a bulging fat pad surrounding the hip suggesting a joint effusion **(A)**. On the initial MRI scans there was a hyper intensity of the left hip and acetabulum (=oedema of the bone marrow) as well as marked joint effusion on the T2 weighted image with fat suppression **(B)**, synovial thickening and enhancement on the post-contrast T1 weighted image with fat suppression **(C)**. These MRI findings were receding following 13 months of anti-TB treatment (T2 weighted image with fat suppression, **D**) on the one hand, but on the other hand a severe narrowing of the joint space, osteophytes and severe deformity of the femoral head became present. These findings of secondary osteoarthrosis were also demonstrated on the follow-up X-ray few months later **(E)**.

A 16 year-old male refugee from Afghanistan with a self-reported unremarkable past medical history presented to our tertiary care hospital complaining of a 3 months history of worsening left-sided hip pain, while otherwise being clinically well without signs of fever. Four weeks prior to presenting to our institution he had been seen in a private surgical practice. An ultrasound of the hip revealed significant effusion and joint fluid aspirate obtained via subsequent puncture was sent for conventional bacterial cultures. The patient was discharged home on ibuprofen; conventional microbiological culture showed no growth of bacteria. On presentation to our A&E department he was unable to walk due to severe left-sided hip pain, while being otherwise clinically well. His routine bloods showed a mildly raised CRP (26 mg/l), normal FBC and chemistry. Conventional radiography and a subsequent MRI scan of the left hip showed blurring of cortical margins of the femoral head and acetabulum, hyper intensity of the left hip and acetabulum (oedema of the bone marrow) and marked joint effusion on the T2 weighted image with fat suppression as well as synovial thickening and enhancement on the post-contrast T1 weighted image (Figures 1A–C). A chest x-ray showed signs of adult-type TB, but the patient did not display any clinical evidence of active pulmonary tuberculosis (data not shown). Hence, this result was not followed up by a CT scan of the chest. In view of the prolonged, non-acute clinical course, and his background of being born and raised in a TB high-endemic country (national incidence, including HIV+TB: 189 per 100.000; source: http://www.who.int/tb/country/data/profiles/en/), work-up for suspected tuberculosis of the hip was initiated. IGRA testing (QuantiFeronGold^®^, Qiagen) was positive; AFBs were seen on microscopy from joint fluid aspirate, subsequently confirmed as *M. tuberculosis* (MTB) using PCR (GeneXpert MTB/RIF^®^, Cepheid) and culture. Since we were able to diagnose this patient on his joint fluid aspirate (microscopy results available on the same day), we deliberately refrained from a synovial biopsy, a more invasive diagnostic approach, generally recommended to obtain the gold standard specimen if joint involvement is suspected in tuberculosis. An HIV test was negative. The patient reported that he had never received any treatment for TB previously. He was started on isoniazid, rifampicin, pyrazinamide, and ethambutol for 2 months, followed by an additional 10 months period on rifampicin and isoniazid. Drug resistance testing showed a fully sensitive MTB isolate. Treatment was well tolerated and during the 12 months course, the patient showed continuous clinical improvement with regular ibuprofen required until month 3 of treatment. During this phase, treatment was supplemented by pantoprazole. Follow-up clinical visits revealed no signs of side effects and radiological assessment documented gradual improvement on MRI imaging but also unavoidable long-term damage to the left hip joint on conventional x-ray in terms of severe narrowing of the joint space, osteophytes and severe deformity of the femoral head was evident (Figures 1D,E). Though the patient was able to move slowly without support and almost free of pain from month 9 of anti-tuberculosis treatment, regaining his full range of movement was impossible due to extensive destruction of the left hip. Thus, almost 2 years following TB diagnosis he underwent hip-replacement surgery and is currently in perfect health having regained full mobility.

## TAM-TB Assay Results

**Figure 2:**
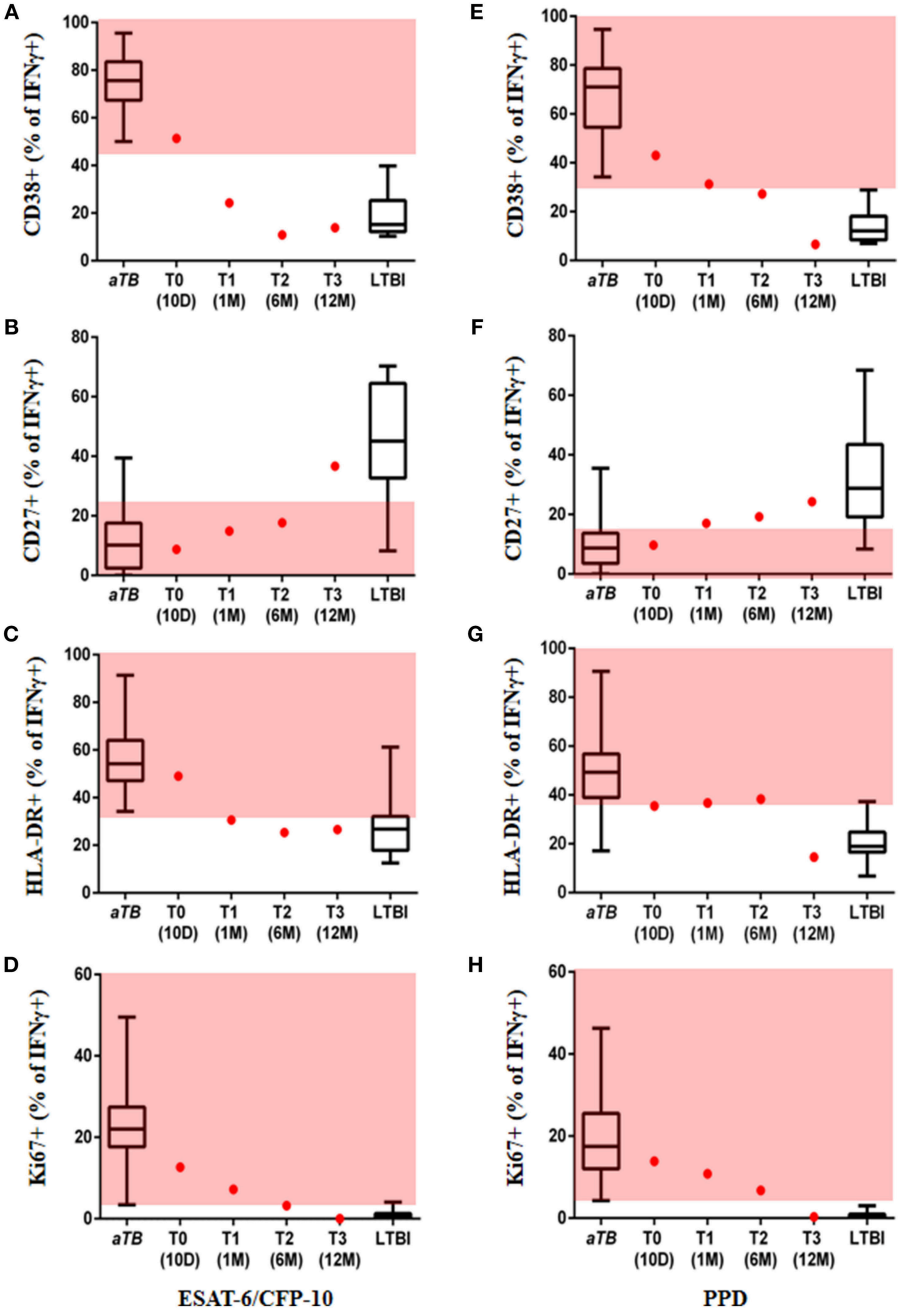
Marker expression on MTB-specific CD4 T cells. Values are provided at the start, during and at the end of treatment. Frequency of ESAT-6/CFP-10 stimulated MTB-specific CD4 T cells expressing the markers CD38 **(A)**, CD27 **(B)**, HLA-DR **(C)**, Ki67 **(D)**, and PPD stimulated MTB-specific CD4 T cells expressing CD38 **(E)**, CD27 **(F)**, HLA-DR **(G)**, Ki67 **(H)** are displayed as red dots at 10 days (T0), 1 month (T1), 6 months (T2), and 12 months (T3) after TB treatment initiation; data from subjects with acute pulmonary tuberculosis and healthy/latent TB infection were included as reference (box and whisker plots). Red area represents the region considered to show an active TB profile with cut-offs based on ROC analysis; ROC cut-off values for ESAT-6/CFP-10, CD38 = 44.9, CD27 = 22.1, HLA-DR = 33.15, Ki67 = 2.43 and for PPD, CD38 = 31.55, CD27 = 18.35, HLA-DR = 35.7, Ki67 = 3.67 (14).

After obtaining written informed consent from the patient, we tested a novel, rapid, sputum independent diagnostic approach, now referred to as the T cell activation marker (TAM)-TB assay. The TAM-TB assay has been shown to accurately differentiate between active pulmonary TB (aTB) and latent TB infection (LTBI) in different age groups by determination of the phenotypic and functional characteristics of MTB-specific CD4 T cells via flow cytometry (10–12); detection of high frequencies of activated (CD38^+^, HLA-DR^+^, and Ki67^+^), CD27^low^ effector memory MTB-specific CD4 T cells is indicative of active TB. Furthermore, results using this approach have been shown to correlate with disease severity and lung tissue destruction (13). Sequential blood samples were obtained from the time of diagnosis (T0, day 10 after treatment initiation), 1 month (T1), 6 months (T2) post-treatment initiation, and at end of treatment (12 months, T3) in order to determine expression levels of the T cell activation markers CD38, HLA-DR, and Ki67 and the maturation marker CD27 on IFNy^+^ MTB-specific CD4 T cells. As previously described (14), the assay was performed by stimulation of PBMCs with ESAT-6/CFP-10, PPD, and Staphylococcal enterotoxin B (SEB), as a positive control, or no added peptide, as a negative control. PBMCs were surface stained with anti-CD4, anti-CD38, anti-CD27, and anti-HLA-DR, fixed and permeabilized, then stained intracellularly with anti-CD3, anti-IFNy, and anti-Ki67. Data from subjects with active pulmonary TB and LTBI from our previous study to define the cut-offs for each marker to differentiate *aTB* and LTBI were used (14). Remarkably, at T0 all four markers assessed in the TAM-TB assay classified our patient as *aTB*, while at T3 they showed a similar phenotype to the healthy IGRA^+^ group of patients, confirming its potential use in the diagnosis of extra-pulmonary TB (Figure 2). For ESAT-6/CFP-10 stimulated cells, the frequency of activated MTB-specific CD4 T cells expressing CD38 and HLA-DR declined rapidly within the first month after treatment initiation from 51.4% at T0 to 24.3% at T1 and from 49 to 31.6%, respectively (Figures 2A,C). Unlike the other two markers (CD27 and Ki67), these markers seem to have provided an LTBI phenotype within a short period (1 month). The frequency of MTB-specific CD4 T cells expressing the cell cycle marker Ki67 showed a gradual decrease and was still within the range observed for *aTB* at T2 post-treatment, but eventually indicated “*LTBI or cure*” at the end of treatment (T3) (Figure 2D). A substantial increase in the frequency of CD27^+^ MTB-specific CD4 T cells was only observed between T2 and T3 (Figure 2D). It is particularly noteworthy that at the end of treatment (T3), the patient showed a TAM-TB assay profile consistent with “*LTBI or cure*” for all four markers. Unlike the ESAT-6/CFP-10 stimulated cells, the frequency of activated PPD stimulated MTB-specific CD4 T cells expressing the activation markers showed the strongest percentage decline between T2 and T3 (Figures 2E–H). Two of the markers (CD27 and CD38) seem to show a borderline LTBI phenotype at T2, with all the markers indicating an “*LTBI or cure*” at the end of treatment (T3) (Figures 2E–H).

Future studies will be needed to determine whether TAM-TB assay results could have guided earlier discontinuation of anti-TB therapy in patients before the defined 12 months period. Of note, an additional TAM-TB result obtained from the patient 2 months after discontinuation of anti-TB treatment (14M) was still consistent with “*LTBI or cure*” (data not shown).

## Discussion/Conclusion

Central and Western Europe, Germany in particular, has seen an increase in TB incidence rate in recent years changing from 5.2/100.000 (2012) to 7.2/100.000 (2015) (15). This change in epidemiology has primarily been attributed to an increase in migration activity from low/intermediate-income, high TB burden settings as a consequence of people fleeing areas of war and devastation. Reactivation of latent tuberculosis infection (LTBI) in the high-income, low TB burden countries has thus become a challenge for clinicians both in adult and pediatric care (16). The adolescent case described in this report is a perfect example of how wildly adapted national diagnostics and treatment guidelines are often limited in the way they are tailored to the local population. Almost any youth of German descent presenting with a painful left hip would have most likely been diagnosed and treated sufficiently according to those guidelines. But pediatric or adolescent tuberculosis, especially in its extra-pulmonary form, is such a rare event in the German setting, that this differential diagnosis was missed initially. It is important to highlight the changes in TB epidemiology and to be aware of TB in patients having moved to central Europe from high TB burden settings. Although a good outcome was achieved following hip replacement, this still remains a problematic long-term solution in someone who is 16 years of age and will inevitably be requiring additional revision-interventions in the future. This again underscores the importance of early accurate diagnosis and treatment.

With this perspective we aim to highlight that the TAM-TB assay is a promising new tool with great potential to improve TB diagnostics, particularly in difficult-to-diagnose cases of extra-pulmonary TB or children and adolescents in whom conventional tests are frequently unsuccessful. Immunological biomarkers have already demonstrated to be very promising to indirectly diagnose TB; and they are highly likely to play an even more prominent role in TB diagnostics in the years and decades to come (3, 17, 18). Several studies have shown the benefit of using flow cytometric techniques to distinguish aTB from LTBI, the earliest reporting the CD27 marker (19). This was followed by a plethora of markers such as HLA-DR, CD38, Ki67, or caspase-3 (12, 20). Other methods using cytokine secretion in whole blood may allow diagnosis of aTB (21), however these methods do not seem to be reliable in monitoring TB treatment (22). Another approach makes use of whole blood transcriptional profiling to identify diagnostic RNA signatures for aTB and LTBI, respectively (2, 23, 24). These studies are extremely interesting and yielded very promising results. However, they have not been shown to be of use for treatment monitoring. Furthermore, until the respective diagnostic signatures have been transformed into a simpler diagnostic test that could be used in daily routine, their use appears impractical due to the highly sophisticated resources required (i.e., running microarrays or RNA sequencing).

Sali et al. was able to show that combined use of the QuantiFeronGold^®^ with a heparin-binding hemagglutinin antigen (HBHA)-based IGRA helped to differentiate Quantiferon-positive children with LTBI from those with aTB (25). While further validation on larger cohorts of MTB-infected children will be necessary to describe the potential of this method for treatment monitoring, issues in identifying asymptomatic children with aTB appears to be its main disadvantage.

In flow cytometry-based assays it has been noted that while CD38, HLA-DR, and Ki67 appeared to be useful markers for monitoring of treatment response in a pulmonary TB cohort; CD27 was a marker that converts rather slowly to its “healthy” state (12, 19). Our assay was successful in diagnosing this case of active extra-pulmonary TB and showed an accurate treatment response at the end of the 12 months treatment period by indicating a “healthy/LTBI” state reflected in respective expression values for all four markers. In addition, all markers (with the exception of HLA-DR in the PPD stimulation assay) did indicate a dynamic trend toward the desired treatment outcome. Whether and how such TAM-TB results during anti-TB treatment could potentially inform decisions on treatment discontinuation in individual patients in the future remains to be evaluated in larger cohorts. Since our patient was 16 years of age and because adolescent children essentially behave like adults, this promising test will also have immediate relevance to adults with extra-pulmonary TB.

However, the TAM-TB assay remains an expensive technique requiring specialized equipment and trained personnel to operate, thus its use as a point of care test (POCT, on-site) technique is currently not feasible. However, several studies have shown that flow-cytometry-based assays can be further simplified by using whole blood rather than PBMC, thus reducing the blood volume required and, in consequence, making them more feasible to use, especially in infants and younger children (26, 27)

Nevertheless, our results provide a first important proof-of-concept that the TAM-TB assay might also be a useful and powerful tool to monitor clinical response to treatment in cases of extra-pulmonary TB. Though the results of this case look promising, they only represent the treatment course of a single patient and one needs to be aware that the respective values of the four TAM-TB markers were referenced to cut-off values obtained from a pulmonary TB cohort. It is likely that since extra-pulmonary TB is a different clinical entity than the pulmonary form of TB, adjusted cut-offs may be required for extra-pulmonary TB cases to allow more accurate interpretation of the results. We do not propose using the TAM-TB assay as a new stand-alone technique for diagnosing tuberculosis, but instead we rather suggest to develop a diagnostic algorithm involving all currently available tools in order to be able to eventually tailor TB treatment duration for individual patients. Still, we are confident that this new assay holds a great potential for the challenging area of TB care and larger prospective clinical trials are currently underway to further validate its performance in the field.

## Author Contributions

MA, UvB, and CG designed and conducted the diagnostic work-up. MA and UvB wrote the manuscript. ID, CZ, and UvB took clinical care of the patient and collected TAM TB samples. MA, KH, and CG ran and analyzed TAM TB assay. JL-Z reported on imaging results and created respective figure for image display.

### Conflict of Interest Statement

The authors declare that the research was conducted in the absence of any commercial or financial relationships that could be construed as a potential conflict of interest.
